# Effects of Mono-(2-Ethylhexyl) Phthalate and Di-(2-Ethylhexyl) Phthalate Administrations on Oocyte Meiotic Maturation, Apoptosis and Gene Quantification in Mouse Model

**DOI:** 10.22074/cellj.2016.4717

**Published:** 2016-09-26

**Authors:** Forouzan Absalan, Sadegh Saremy, Esrafil Mansori, Mahin Taheri Moghadam, Ali Reza Eftekhari Moghadam, Razie Ghanavati

**Affiliations:** 1 Department of Anatomical Science, Faculty of Medicine, Ahvaz Jundishapur University of Medical Sciences, Ahvaz, Iran; 2 Cellular and Molecular Research Center, Faculty of Medicine, Ahvaz Jundishapur University of Medical Sciences, Ahvaz, Iran; 3 Department of Molecular Biology and Development, Faculty of Medicine, Kazerun Islamic Azad University, Kazerun, Iran

**Keywords:** Oocyte Maturation, Apoptosis, Gene Expression

## Abstract

**Objective:**

Phthalates, which are commonly used to render plastics into soft and flexible materials, have also been determined as developmental and reproductive toxicants in human and animals. The purpose of this study was to evaluate the effect of mono-(2- ethylhexyl) phthalate (MEHP) and di-(2-ethylhexyl) phthalate (DEHP) oral administrations on maturation of mouse oocytes, apoptosis and gene transcription levels.

**Materials and Methods:**

In this experimental study, immature oocytes recovered from Naval Medical Research Institute (NMRI) mouse strain (6-8 weeks), were divided into seven different experimental and control groups. Control group oocytes were retrieved from mice that received only normal saline. The experimental groups I, II or III oocytes were retrieved from mice treated with 50, 100 or 200 µl DEHP (2.56 µM) solution, respectively. The experimental groups IV, V or VI oocytes were retrieved from mouse exposed to 50, 100 or 200 µl MEHP (2.56 µM) solution, respectively. Fertilization and embryonic development were carried out in OMM and T6 medium. Apoptosis was assessed by annexin V-FITC/Dead Cell Apoptosis Kit, with PI staining. In addition, the mRNA levels of *Pou5f1, Ccna1* and *Asah1* were examined in oocytes. Finally, mouse embryo at early blastocyst stage was stained with acridine-orange (AO) and ethidium-bromide (EB), in order to access their viability.

**Results:**

The proportion of oocytes that progressed up to metaphase II (MII) and 2-cells embryo formation stage was significantly decreased by exposure to MEHP or DEHP, in a dose-dependent manner. Annexin V and PI positive oocytes showed greater quantity in the treated mice than control. Quantitative reverse transcriptase-polymerase chain reaction (qRT-PCR) revealed that expression levels of *Pou5f1, Asah1* and *Ccna1* were significantly lower in the treated mouse oocytes than control. The total cell count for blastocyst developed from the treated mouse oocytes was lower than the controls.

**Conclusion:**

These results indicate that oral administration of MEHP and DEHP could negatively affect mouse oocyte meiotic maturation and development *in vivo*, suggesting that phthalates could be risk factors for mammalians’ reproductive health. Additionally, phthalate-induced changes in *Pou5f1, Asah1* and *Ccna1* transcription level could explain in part, the reduced developmental ability of mouse-treated oocytes.

## Introduction

Decline in human and animal fertility, mainly caused by environmental chemicals, desires more attentions of scientific communities as well as general public (1). Meanwhile, phthalates, as a group of artificial organic chemicals, are generally used to give softness and flexibility to plastics. Regarding the massive applications in laboratory and medical products, slow release into the nature, inability for entering into the environmental cycles and exposing to human body (2, 3), phthalates raise the concern on the potential health side-effects.

The di-(2-ethylhexyl) phthalate (DEHP) is a high production volume (HPV) chemical, leaching of which in environment causes adverse effects on reproduction and development. In several species, DEHP are hydrolyzed into specific hydrolytic monoesters in mouth, skin, stomach, intestine or blood, called mono-(2-ethylhexyl) phthalate (MEHP). It is reported that MEHP could be an active compound, reliably demonstrating many effects of DEHP *in vivo* (4).

Investigations indicated that exposing to phthalates could lead to birth defects, asthma, neuro-developmental problems in newborns as well as obesity or infertility (5). It has been demonstrated the critical adverse effects of phthalates on male or female reproductive systems by interfering with production of testosterone or estradiol, respectively (6). Thus far, very few studies have examined the potential effect of phthalates on ovarian follicles *in vivo*, as a target site of this chemical agent.

It has been shown that DEHP exposure in animal and culture models could decrease estradiol level due to reduced aromatase expression, prolonged estrous cycles, ovulation defect and ovarian deterioration (7). In 2003, Anas et al. (8) revealed that exposing to MEHP *in vitro* inhibited meiotic maturation of bovine oocytes in a dose-dependent manner. This finding was subsequently validated on mouse oocytes, where Shahverdi and colleagues showed that oral MEHP administration prohibited meiotic maturation in the mouse embryos and oocytes (9). In addition, investigations on zebrafish exhibited deleterious effects of DEHP on the particular molecular biomarkers of oogenesis and fetal development (10). In that respect, findings revealed that both DEHP and MEHP inhibited follicle growth in mice by reducing estradiol production in antral follicles (4).

It has currently been proved that fully mature mammalian oocyte, preserving maternal genetic information, is transcriptionally silent and use transcripts which is only synthesized and accumulated through early development (11). Therefore, determining the gene expression profiles that occur during the oocyte development and progression of a fertilized egg is necessary (12). In line with that, mRNA expression level of particular genes was investigated in bovine oocytes exposed to phthalates. Findings of this study showed reduced mRNA expression levels of *Pou5f1* (pluripotency factor), *Asah1* (an anti-apoptotic maker) and *Ccna2* (involved in cell cycle control) due to the exposure of MEHP into mature oocytes (13).

In current study, we determined the effect of DEHP or MEHP oral administration on oocyte meiotic maturation and apoptosis. We also estimated the *Pou5f1, Ccna1* and *Asah1* transcription levels by real-time polymerase chain reaction (RT-PCR) in metaphase II (MII) stage of oogenesis. In addition, blastocyst quality was evaluated by acridine-orange (AO) and ethidium-bromide (EB) staining.

## Materials and Methods

### Immature oocyte retrieval

In this experimental study, 210 healthy adult female Naval Medical Research Institute (NMRI) mice (6-8 weeks old, 20-30 g) were obtained from Jundishapur University Experimental Research Center. The animals were housed under standard laboratory conditions with 12 hours dark and 12 hours light cycles, relative humidity of 50 ± 5% and temperature of 22 ± 3˚C. The mice were divided into six groups and orally administrated 50, 100 or 200 μl of 2.56 μM DEHP or MEHP solution (1 μl DEHP or 0.7 μl MEHP dissolved in Dimethyl Sulfoxide (DMSO, 0.1%, respectively) (9) for 15 days. Subsequently, the mice were sacrificed by spinal dislocation, followed by dissection under sterile conditions and collection of ovary tissues. Next, these tissues were preserved in 500 μl drops of Minimum Essential Medium Eagle-Alpha (MEM-α) culture media, containing 5% fetal calf serum (FCS). Cumulus oocyte complexes (COCs) were aspirated from follicles using insulin syringe. COCs with at least three layers of cumulus surrounding a homogeneous cytoplasm were selected for each group. All procedures were ethically performed in accordance with Jundishapur University (Ahvaz, Iran) animal scientific procedure acts.

### In vitro maturation

COCs, were washed three times in phosphate-buffered saline (PBS) and transferred to 50 μl droplets of ovarian maturation medium (OMM) overlaying with mineral oil. The COC-containing droplets were incubated in humidified air condition with 5% CO_2_ for 22 hours at 38.5˚C. This experiment was evaluated on the control (contains 131 normal saline treated oocytes) and six experimental groups. The experimental groups I, II and III were respectively composed of 101, 112 and 119 oocytes treated with 50, 100 and 200 μl of DEHP (2.56 μM, Sigma-Aldrich Laborchemikalien GmbH, Germany). In contrast, the experimental groups IV, V and VI were respectively comprised of 107, 114 and 124 oocytes exposed to 50, 100 and 200 μl of MEHP (2.56 μM, Wako Chemical GmbH, USA). *In vitro* maturation procedures were evaluated by inverted microscope after denuding the cumulus cells in hyaluronidase (1.000 U/ml, Sigma-Aldrich, USA) by gentle vortexing. In this part, oocytes without any change in their nuclei were considered as germinal vesicle (GV) or immature; those with nuclear breakdown were evaluated as GV breakdown (GVBD), while the oocytes with meiotic sings and polar bodies were deemed as mature or MII oocytes.

### In vitro fertilization and development of matured oocytes

The epididymis tails of sacrificed male NMRI mice were dissected and placed into 500 μl drops of T6 media containing 5 mg/ml bovine serum albumin (BSA, Gibco, USA). After incubation at 37˚C temperature and 5% CO_2_ concentration for 1.5 hours, the active and normal sperms were collected and transferred along with oocytes into drops of T6 media, containing 16 mg/ml BSA. Following 4-6 hours incubation, MII oocytes were transferred into a new medium condition, containing T6 with 5 mg/ml BSA. Ultimately, the percentage of oocytes cleaved to twoor four-cells stage was assessed at 42-44 hours post-fertilization. In addition, the average number of developed embryos up to the blastocyst stage was assessed on days 7-8 of the post-fertilization by inverted microscope (Olympus, Japan).

### Annexin V staining

Apoptosis was determined using Annexin V-FITC/propidium iodide (PI) apoptosis detection kit (Beyotime Institute of Biotechnology, China). As a phospholipid-binding protein, Annexin V-FITC has a strong affinity to phosphatidyl serine (PS) on the membrane of early-apoptotic cells. On the other hand, PI can permeate the membrane of late-apoptotic and necrotic cells and stain the nuclei. In this experiment, the mouse oocytes that exposed with 50, 100 or 200 μl of MEHP or DEHP were cultured for 22 hours in OMM, followed by denudation in 1,000 U/ml of hyaluronidase. They were subsequently washed twice with PBS and suspended in 200 μl binding buffer (Beyotime Institute of Biotechnology, China). Afterwards, 5 μl Annexin V-FITC and 10 μl PI were added to 100 μl resultant cell suspensions and incubated for 15 minutes at room temperature protecting from light exposure. Next, 400 μl PBS was added to those cells and the proportion of apoptotic cells were analyzed by FC500 flow-cytometer (Beckman coulter, USA). This experiment was performed for 114 oocytes in control group, as well as 98, 102, 110, 120, 101 and 117 oocytes for experimental groups I-VI, respectively.

### Reverse transcription polymerase chain reaction quantification

In this study, we relatively compared the mRNA expression level of three genes (*Pou5f1, Ccna1* and *Asah1*) to a housekeeping gene (Gapdh), using quantitative RT-PCR (qRT-PCR). To do so, putative arrested MII stage mouse oocytes, exposed to 100 μl MEHP (n=112) or DEHP (n=123), were collected after 22 hours of *in vitro* maturation and cumulus cells were denuded in hyaluronidase (1,000 U/ml) by gentle vortexing. The experiments were performed in at least three biological repeats. All collected samples were washed in PBS, snap frozen in liquid nitrogen and stored at -80˚C until RNA extraction.

Using the RNX-Plus^TM^ (Fermentase, Germany), RNA was extracted from samples according to the manufacturer’s instruction. RNA concentration was then determined by UV spectrophotometry (Eppendorff, Germany). To eliminate genomic contamination, RNA was treated with DNase I kit (EN0521, Fermentase, Germany). The cDNA was produced from RNA samples using RevertAid^TM^ first strand cDNA synthesis kit (Fermentas, Germany) based on the manufacturer’s instruction. Primers were designed by using the NCBI website (Table 1) and synthesized by Cinnagen Company (Iran). PCR reactions were performed using SYBER Green master mix (Applied Biosystems, USA), cDNA samples and individual primer sets. QRT-PCR program was started with an initial melting cycle for 5 minutes at 95˚C to activate the polymerase, followed by 40 cycles of melting (10 seconds at 95˚C), annealing (15 seconds at 60˚C) and extension (30 seconds at 72˚C). The quality of the qRT-PCR reactions were confirmed by melting curve analysis. The efficiency of individual gene mRNA expression was determined by using the standard curve. Relative quantification levels were identified using 2^-ΔΔCt^ method.

**Table 1:** List of the primers utilized for qRT-PCR experiment


Gene	Primer sequences (5ˊ-3ˊ)

*Pou5f1*	F: AGAGGGAACCTCCTCTGAGC
	R: CCAAGGTGATCCTCTTCTGC
*Ccna1*	F: CGCACAGAGACCCTGTACTT
	R: TTGGAACGGTCAGATCAAAT
*Asah1*	F: TAACCGCAGAACACCGGCC
	R: TTGACCTTTGGT
*Gapdh*	F: TGCAGTGCCAGCCTCGTG
	R: TTGATGGCAACAATCTCCACTT


QRT-PCR; Quantitative reverse transcription polymerase chain reaction.

### Acridine-orange and ethidium-bromide staining of blastocyst

AO and EB staining are generally appropriate candidates for evaluation of cell viability. Using this method, the live cells show a green fluorescence color due to the AO staining, while dead cells represent an orange fluorescence upon EB staining. In this experiment, a stock solution was prepared by dissolving 50 mg EB and 15 mg AO (both purchased from Sigma-Aldrich, USA) in 50 ml of ethanol 2% (1 ml ethanol 95% diluted in 49 ml distilled water). The stock solution was divided into 1 ml aliquots and preserved at -20˚C temperature. Each aliquot was later diluted in 1x PBS, and preserved in dark glass tubes at 4˚C. For embryo staining, 25 μl of the prepared master-mix was added to each sample spread on the slide. The slides were ultimately observed by fluorescence microscope (Olympus, Japan) with a 515 nm filter .

### Statistical analysis

All data were analyzed using one-way repeated measure analysis of variance (ANOVA) followed by Tukey’s post hoc test with a significance threshold of P<0.05. The statistics software package SPSS Version 16.0 was used to perform the calculations. The error-bars were represented as mean ± SD.

## Results

### Mono-(2-ethylhexyl) phthalate and di-(2-ethylhexyl) phthalate influences on oocyte development

The effects of DEHP or MEHP exposure on oocyte development were assessed *in vitro* by comparing several doses of these phthalates with control group (Table 2). Findings showed that exposing to DEHP or MEHP, in dose-dependent manner, led to significantly reduced frequency of the oocytes progressing into MII stage. In addition, both, DEHP and MEHP, had a deleterious carrying-over effect on the oocyte developmental competence, reflected by decrease in the percentage of developing blastocysts in the treated groups, compared to the control (P<0.05). Further analyses revealed that oocytes development percentage at the maturation MII stage was significantly decreased with exposure to 50, 100 or 200 μl DEHP (42.8, 36.2 or 26.5%, respectively) in the treated mice compared to the controls (67.37%). This frequency was significantly lower in 200 μl DEHP treatment compared to the other concentrations. In contrast to the control (67.37%), exposure of 50, 100 or 200 μl MEHP (2.56 μm) for 22 hours in vitro demonstrated respectively a frequency of 37.8, 27.6 or 17.3% for the MII stage oocytes (P<0.05). Similar to DEHP, the lowest rate of MII stage oocytes proportion was observed in 200 μl MEHP exposed cells, compared to the other treatments (P<0.05). In addition, the rate of oocytes developing 2-4 cells stage embryo and blastocyst stage was lower in all experimental groups than control. The oral administration of DEHP or MEHP effectively decreased the percentage of embryo formation in comparison to the control. Comparing MEHP and DEHP experimental groups showed deleterious effects of MEHP on developmental competence of oocyte and embryo up to blastocyst stage.

### Effects of mono-(2-ethylhexyl) phthalate and di-(2-ethylhexyl) phthalate on oocyte apoptosis/necrosis/health

**Fig.1:**
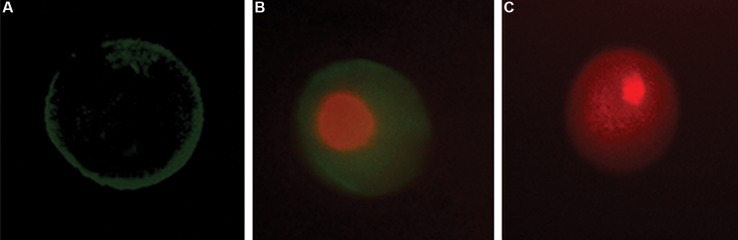
The Representative image of the oocyte staining with propidium iodide (PI) and Annexin V-FITC. A. Early apoptotic, B. Late apoptotic, and C. Necrotic mouse oocytes using PI and Annexin V-FITC staining.

Following the Annexin V-FITC staining, denuded oocytes were classified into four groups: i. Necrotic cells with PI-positive red nuclei and cytoplasm, ii. Early apoptotic cells with homogeneous Annexin V-positive signals in membrane, iii. Eate apoptotic cells with PI-positive nuclei, and iv. Normal cells which is not stained by Annexin V-FITC or PI (Fig .1).

**Fig.2:**
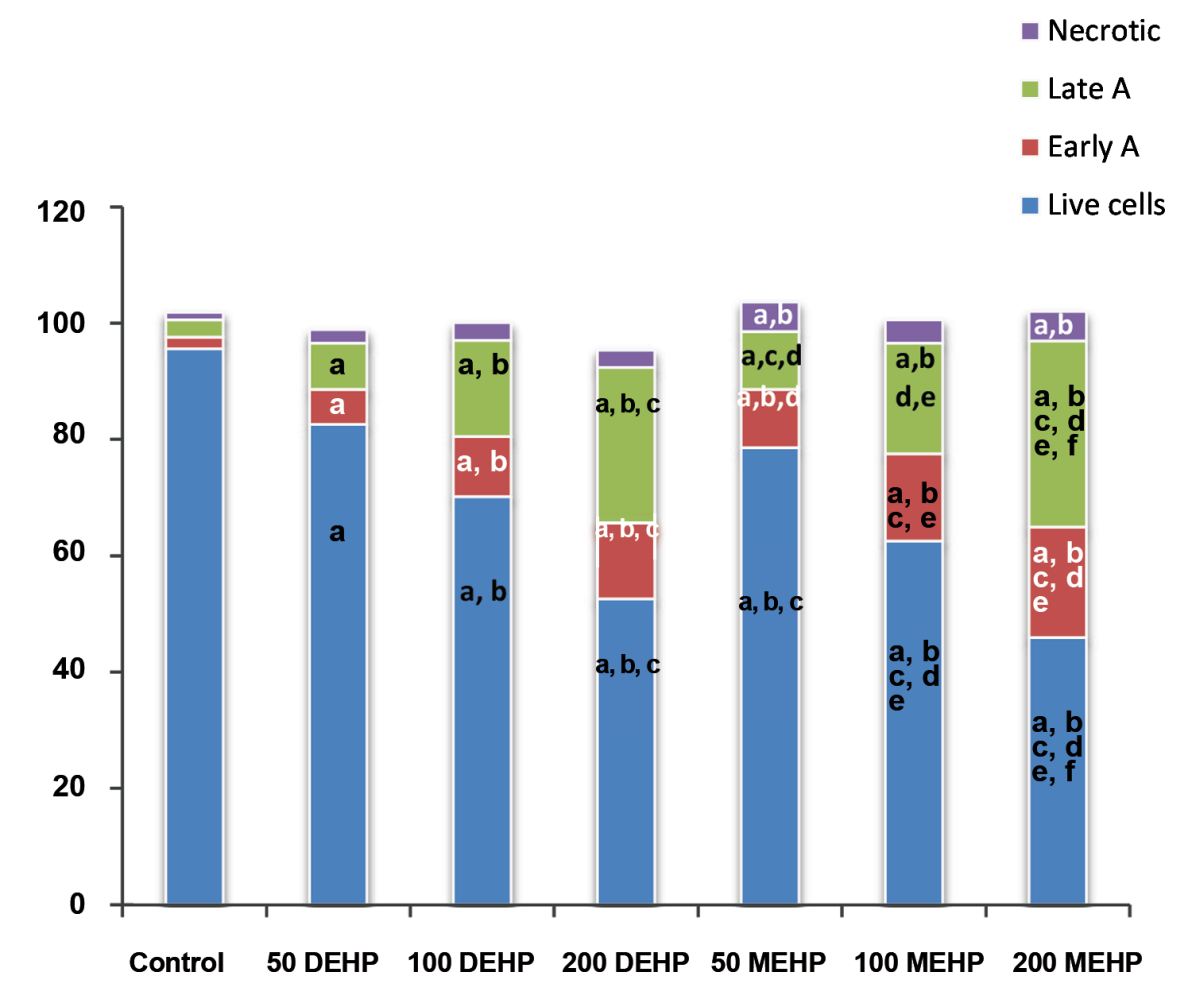
Image represents frequency of the live, early apoptotic, late apoptotic and necrotic oocytes (percentages) in experimental and control groups. Early A; Early apoptosis, Late A; Late apoptosis, MEHP; Mono-(2-ethylhexyl) phthalate, DEHP; Di-(2-ethylhexyl) phthalate, a; Significant difference with control group, b; Significant difference with experimental group-I (50 μl DEHP), c; Significant difference with experimental group-II (100 μl DEHP), d; Significant difference with experimental group-III (200 μl DEHP), e; Significant difference with experimental group-IV (50 μl MEHP), and f; Significant difference with experimental group-V (100 μl MEHP).

Analysis of the oocyte groups (Fig .2), exposed to DEHP, showed a dose-dependent (from low to high concentration) increase in early and late apoptosis, 14, 26.9 and 39.8%, respectively (P<0.05). In contrast, findings showed significantly lower normal oocytes in experimental groups treated with DEHP, compared to the control. No difference was observed between different groups, for necrotic cell staining (Fig .2). In comparison with DEHP, the effect of MEHP on the oocytes was less reversible with no dose-dependency manner. Early apoptosis in mouse oocytes exposed to 50 or 100 μl MEHP was significantly increased in contrast to mouse oocytes exposed to similar concentration of DEHP (Fig .2). Whereas, oral administration of the MEHP (200 μl) can affect early and late apoptotic oocytes compared to 200 μl DEHP treated mice. Necrotic oocytes in all exposed MEHP groups showed a significant difference rather than control (5, 4 and 5 vs. 1.3%, respectively).

**Table 2:** Analysis of *in vitro* maturation (IVM) and fertilization (IVF), after oocyte culturing in experimental and control groups


Stages of development			Groups	Stages of development			Groups
96 hours after IVF	MII	48 hours after IVF		96 hours after IVF	MII	48 hours after IVF	

21.6 ± 1	37.6 ± 1.1	67.3 ± 2	Control (n=131)	21.62 ± 1	37.6 ± 1.1	67.3 ± 2	Control (n=131)
5.5 ± 0.3^a, b^	18.37 ± 0.4^a^	37.87 ± 0.9^a^	Exp IV 50 μL MEHP (n=107)	8.6 ± 0.3^a^	20.37 ± 0.7^a^	41.87 ± 1^a^	Exp I 50 μL DEHP (n=101)
2.5 ± 0.3^a, c, e^	10.6 ± 0.4^a, c, e^	27.62 ± 0.9^a, c, e^	Exp V 100 μL MEHP (n=114)	5.2 ± 0.3^a, b^	14.12 ± 0.3^a, b^	36.22 ± 1^a^	Exp II 100 μL DEHP (n=112)
0.8 ± 0.2^a, d, e^	6.7 ± 0.3^a, e, f^	19 ± 0.01^a, e, f^	Exp VI 200 μL MEHP (n=124)	1.3 ± 0.3^a, b, c^	8.6 ± 0.5^a, b, c^	26.5 ± 0.8^a, b, c^	Exp III 200 μL DEHP (n=119)


Exp; Experiment, ^a^; Significant differences with control group, P<0.05, ^b^; Significant differences with experimental group I in the same column and row, P<0.05, ^c^; Significant difference with experimental group II in the same column and row, P<0.05, ^d^; Significant difference with experimental group III in the same column and row, P<0.05, ^e^; Significant difference with experimental group IV in the same column and row, P<0.05, ^f^; Significant difference with experimental group V in the same column and row, P<0.05, MEHP; Mono-(2-ethylhexyl) phthalate and DEHP; Di-(2-ethylhexyl) phthalat.

### The effect of mono-(2-ethylhexyl) phthalate and di-(2-ethylhexyl) phthalate on maternal mRNA expression level

**Fig.3:**
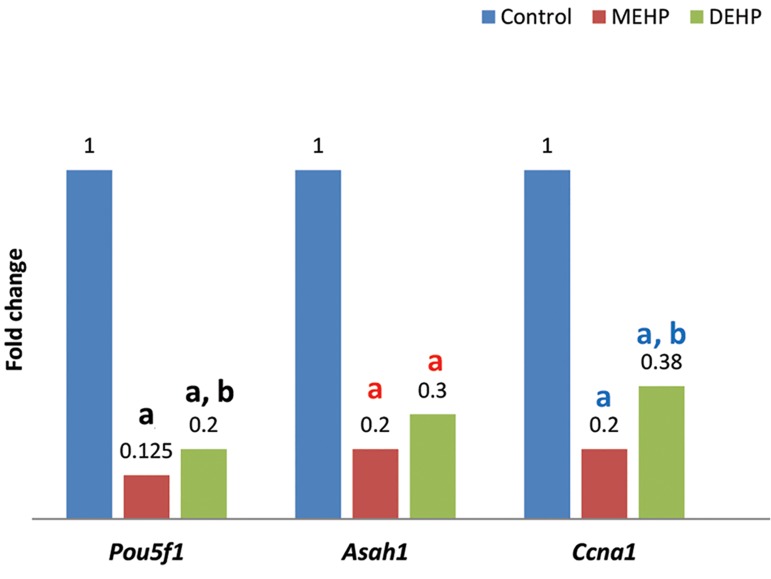
mRNA expression level analysis of *Pou5f1, Asah1* and *Ccna1* in control group and oocytes exposed to 100 μl of MEHP or DEHP. MEHP; Mono-(2-ethylhexyl) phthalate, DEHP; di-(2-ethylhexyl) phthalate, a; Significant difference of control group and DEHP, P<0.05 and b; Significant difference of MEHP group and DEHP, P<0.05.

Real-time PCR analysis revealed significantly reduced level of *Ccna1, Pou5f1,* and *Asah1* mRNA expression in the mouse oocytes, exposed to 100 μl MEHP or 100 μl DEHP (2.56 μM) compared to the control group (Fig .3). Further analyses demonstrated that mRNA level of *Ccna1* and *Pou5f1* genes were significantly lower in the mouse oocytes exposed by 100 μl MEHP rather than 100 μl DEHP. Although lower Asah1 mRNA expression was also observed in oocytes exposed by MEHP, no significant difference was observed in contrast to DEHP.

### Evaluation of the survived and dead blastomeres in developing blastocysts

**Fig.4:**
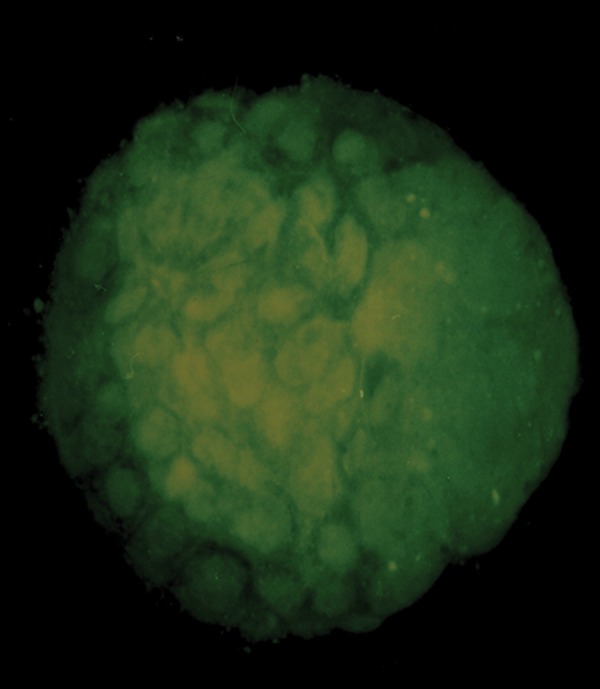
Representative image of the blastocyst staining with AO and EB. AO; Acridine-orange and EB; Ethidium-bromide.

Ultimately, out of three repeats, one or two blastocyst(s) was selected from each experimental and control group to analyze the rate of dead and survived blastomers by using AO and EB staining (Fig .4). Findings showed that the rate of living blastomeres per embryo was significantly lower while the oocytes were exposed to MEHP or DEHP (47.9 ± 5.01, 56.66 ± 5.4) compared to the control group (84.1 ± 7.06, Table 3). The proportion of dead cells per embryo tended to be higher in the MEHPor DEHP-treated embryos relative to controls (P<0.05, Table 3). In addition, analyses of the blastomeres presented significantly different proportions of survived and dead cells in the MEHP-treated cells compared to DEHP.

**Table 3:** Comparison of live and dead blastomeres stained in control group and the cells exposed to 100 μl MEHP or 100 μl DEHP


Groups	Number of staining blastocysts	Mean of Live blastomeres ± SD	Dead cells

Control	4	84.1 ± 7.06	0
MEHP	3	47.9 ± 5.01^a^	3.4 ± 0.55^a^
DEHP	3	56.66 ± 5.4^a, b^	2.67 ± 1.37^a, b^


MEHP; Mono-(2-ethylhexyl) phthalate, DEHP; Di-(2-ethylhexyl) phthalate, ^a^; Significant difference of control and MEHP-treated groups, P<0.05, and ^b^; Significant difference of DEHP and MEHP-treated groups, P<0.05.

## Discussion

Releasing the environmental toxicants -such as phthalatesinto the air, soil and surface water has led to serious health hazards for human and animals. Thus these factors could affect embryonic development and reproductive organs (1, 14, 15). The current study provides evidences that oral administration of phthalates can impairs the meiotic and developmental competence of mouse oocytes.

This experiment showed that DEHP or MEHP induction suppressed oocyte development at all examined concentrations in a dose-dependent manner, after gavage administration. It has been demonstrated that DEHP, as peroxisome proliferator, has specific effects on estradiol production by inhibiting follicle stimulating hormone (FSH)- stimulated cAMP accumulation in rat granulosa cells (16, 17). Mechanistically, previous studies revealed that DEHP, through its metabolite MEHP, aberrantly regulates peroxisome proliferator activated receptor (PPAR)-mediated signaling pathways, leading to suppressing transcription of aromatase (P450Arom) as well as estradiol production, independent of cAMP stimulation in the ovary (18, 19).

PPARs play essential roles in the management of cellular differentiation, development, metabolism and tumorigenesis at higher organisms. It has been suggested that PPARs (20) could interrupt the growth time and follicular differentiation by inducing enzymatic free radical and oxidative stress pathways (21). Regarding these data, conversion of DEHP to the MEHD is proposed as a crucial point for PPAR activation and toxicological effects (18). In this experiment, we showed that MEHP bears more toxic effect rather than DEHP. In competence with this finding, evidences showed that MEHP cytotoxicity was 10-fold more than its parent compound (DEHP), due to the activity of MEHP (22, 23). It was suggested that MEHP, compared to DEHP, induces oxidative stress by suppressing different antioxidant enzymes (24). Therefore, it is most likely important to develop an approach to inhibit the ability of body in converting DEHP into MEHP. In addition, structural differences between DEHP and MEHP may lead to their distinct effects on antioxidant enzymes. DEHP, through lipophilic feature caused by two 2-ethylhexanol branched chains, can activate intracellular signal cascades while cross the lipid membrane. In contrast, MEHP has only one 2-ethylhexanol branched chain and consequently less lipophilic feature than DEHP. It has been demonstrated that MEHP affects the signaling molecules located on membrane, instead of activating intracellular molecules (20).

Our findings, based on Annexin V/PI staining, implicated that exposure to MEHP or DEHP might cause oocyte death by induction of apoptotic signaling pathway. However, further investigations are required to determine mechanisms involved in apoptosis, due to oral phthalate esters induction.

On the other hand, with regards to the study indicating that regulation of maternal mRNA expression in oocytes could direct maturation procedure (28), we evaluated the phthalate effects on this procedure via analysis of mRNA expression level. In particular, we showed reduced level of *Pou5f1, Asah1* and *Ccna1* mRNA expressions in the experimental groups, suggesting that phthalate might influence the quality of developed embryo by regulating particular gene transcriptions. *Pou5f1* is one of the maternal regulated genes, playing critical role in defining totipotency and inducing pluripotency of embryonic cells (29). Upor down-regulation of *Pou5f1* can alter cell signaling, epigenetic fate, transcriptional and post-transcriptional regulation, cell cycle behavior and apoptosis during early embryonic development (30-32). In current study, reduced level of *Pou5f1* expression could be a likely cause of decreased developmental competence in treated oocyte groups.

It has been shown that Pou5f1 activity could cause blastocyst developmental arrest through mitochondrial dysfunction and induction of apoptosis. In current experiment, AO and EB staining of blastocysts, derived from treated mouse oocytes, indicated decreased embryo quality and increased death incidence in embryonic cells.

Consistent to this, Gendelman and Roth (33) showed that thermal stress reduced transcription of *Pou5f1* before further expression of embryonic genome activation at the blastocyst stage. It is proposed that impairment of Pou5f1, led by exposure to MEHP or DEHP, could be intensified in later developmental stages, subsequently culminating in reduced frequency of developed embryos into the blastocyst stage.

It is has been approved that genetic programs, contributing to cell division control, play critical role on transformation of meiotic into mitotic cell cycle upon fertilization. Meanwhile, cell cycle progression is regulated by various cyclin activities (33). *Ccna1* encodes cyclin A1 which is involved in DNA double strand break repair and regulation of metaphase in meiotic cell cycles (34, 35). Previous studies have reported expression of cyclin A1 in testis (36-39). In addition, there are evidences implicating on the expression of this cyclin in ovary/oocyte (36). Fuchimoto et al. (39) showed that cyclin A1 was expressed in oocytes as well as single cell embryos. Interestingly, our findings indicated that cyclin A1 was expressed in mature oocytes. It was suggested that expression of cyclin A1 in oocytes is part of normal role of this gene (34). Thus, reduced Ccna1 mRNA expression level in MEHP or DEHP treated oocytes seems to explain the reduced normal function of this gene, through oogenesis stages. Down-regulation of these gene expressions, by exposing to the other endocrine disruptors like 4-nonylphenol, could lead to defect of cyclin A and cyclin B1 during maturation (39, 40).

Asah1 mRNA encodes acid ceramidase (AC), an enzyme required for early embryo survival, by maintaining a balance between ceramide (a pro-apoptotic lipid) and an anti-apoptotic lipid named sphingosine-1-phosphate (S1P). It has been shown that AC has important role in ceramide-related changes, leading to cell cycle arrest and/or death (41). Eliyahu et al. (42) revealed that AC depletion, during follicular transition from secondary to antral stages, led to apoptosis of oocytes. Here, we reported phthalate-induced reduction in Asah1 transcription levels in MII-stage oocytes. This finding might associate with an increased number of annexin V-positive oocytes, presumably due to an increase in ceramide levels. Moreover, the reduced transcription level of Asah1 mRNA might explain the decreased percentage of cleaved embryos, leading to further development towards blastocyst stage as well as increased proportion of cell death in the blastocysts. Investigations proposed that exposure to DEHP causes apoptosis through activation of PPARs pathway (43). Moreover, it has been suggested that PPARs are the mediators of phthalate-induced alterations in the male and female reproductive tract (44); therefore, PPAR activation could be considered as a critical process at this experiment. Recent reports have also shown that S1P, a downstream product of AC activity, can prevent apoptosis in the ovaries of several species (42). All of these findings are in agreement with our hypothesis suggesting that AC has a major protective function during oocyte and follicle development.

## Conclusion

Ultimately, our investigations on the models *in vivo* indicated that phthalates had a deleterious carrying-over effect on oocyte developmental competence, reflected by a reduced proportion of oocytes undergoing maturation, fertilization, cleavage, and further development towards the blastocyst stage. The reduced developmental competence of MEHP-treated oocytes was strongly associated with alterations in the levels of *Ccna1, Asah1,* and *Pou5f1* mRNA expression. This study revealed that exposure to the environmental concentrations of phthalate affected the ovarian pool of oocytes and these effects persisted into the later stage. However, phthalate-induced alterations in oocyte maternal mRNA may highlight the risk association with exposure of animals to environmental contaminations and their potential to compromise fertility.
